# Clinical Validation of a Deep Learning-Based Software for Lumbar Bone Mineral Density and T-Score Prediction from Chest X-ray Images

**DOI:** 10.3390/diagnostics14121208

**Published:** 2024-06-07

**Authors:** Sheng-Chieh Tseng, Chia-En Lien, Cheng-Hung Lee, Kao-Chang Tu, Chia-Hui Lin, Amy Y. Hsiao, Shin Teng, Hsiao-Hung Chiang, Liang-Yu Ke, Chun-Lin Han, Yen-Cheng Lee, An-Chih Huang, Dun-Jhu Yang, Chung-Wen Tsai, Kun-Hui Chen

**Affiliations:** 1 Department of Orthopedic Surgery, Taichung Veterans General Hospital, Taichung 40705, Taiwan; 2 Rong Hsing Research Center for Translational Medicine, National Chung Hsing University, Taichung 402202, Taiwan; 3 PhD Program in Translational Medicine, National Chung Hsing University, Taichung 402202, Taiwan; 4 Acer Medical Inc., 7F, No. 86, Sec. 1, Xintai 5th Rd. Xizhi, New Taipei City 221421, Taiwan; 5 Department of Post-Baccalaureate Medicine, College of Medicine, National Chung Hsing University, Taichung 402202, Taiwan; 6 Graduate Institute of Biomedical Engineering, National Chung Hsing University, Taichung 402202, Taiwan; 7 Department of Computer Science and Engineering, National Chung Hsing University, Taichung 402202, Taiwan; 8 Acer Inc., 7F-5, No. 369, Fuxing N. Rd., Songshan Dist., Taipei City 10541, Taiwan; 9 Joy Clinic, No. 37 Jilin Rd., Luzhu Dist., Taoyuan City 338120, Taiwan; 10 Department of Computer Science and Information Engineering, Providence University, Taichung 40301, Taiwan

**Keywords:** osteoporosis, bone mineral density (BMD), chest X-ray, artificial intelligence, deep learning

## Abstract

Screening for osteoporosis is crucial for early detection and prevention, yet it faces challenges due to the low accuracy of calcaneal quantitative ultrasound (QUS) and limited access to dual-energy X-ray absorptiometry (DXA) scans. Recent advances in AI offer a promising solution through opportunistic screening using existing medical images. This study aims to utilize deep learning techniques to develop a model that analyzes chest X-ray (CXR) images for osteoporosis screening. This study included the AI model development stage and the clinical validation stage. In the AI model development stage, the combined dataset of 5122 paired CXR images and DXA reports from the patients aged 20 to 98 years at a medical center was collected. The images were enhanced and filtered for hardware retention such as pedicle screws, bone cement, artificial intervertebral discs or severe deformity in target level of T12 and L1. The dataset was then separated into training, validating, and testing datasets for model training and performance validation. In the clinical validation stage, we collected 440 paired CXR images and DXA reports from both the TCVGH and Joy Clinic, including 304 pared data from TCVGH and 136 paired data from Joy Clinic. The pre-clinical test yielded an area under the curve (AUC) of 0.940, while the clinical validation showed an AUC of 0.946. Pearson’s correlation coefficient was 0.88. The model demonstrated an overall accuracy, sensitivity, and specificity of 89.0%, 88.7%, and 89.4%, respectively. This study proposes an AI model for opportunistic osteoporosis screening through CXR, demonstrating good performance and suggesting its potential for broad adoption in preliminary screening among high-risk populations.

## 1. Introduction

Osteoporosis is a chronic skeletal disease characterized by low bone mineral density (BMD) and microarchitectural deterioration of bone tissue, leading to more porous bone and an increased risk of fractures [1,2,3,4]. It is often referred to as a “silent disease” as there are typically no symptoms until a fracture occurs [2,5]. Fractures associated with osteoporosis, particularly spine and hip fractures, impose a substantial burden on healthcare systems due to hospitalization, long-term care, and disability [2,6,7]. Worldwide, osteoporosis is a major public health concern with a growing prevalence due to the aging population [8]. It affects a significant portion of the population, particularly postmenopausal women and older adults [5,9]. In Taiwan, data from the Nutrition and Health Survey in Taiwan (NAHSIT 2004–2008) indicate a concerning statistic of osteoporosis, with a prevalence of 22.57% in men and 41.17% in women over 50 years old when osteoporosis is defined as having at least one of the lumbar spine, femoral neck, or forearm meeting the diagnostic criteria [10]. Despite this, screening rates remain suboptimal, partly due to the lack of awareness about the disease and accessibility issues [11].

Osteoporosis screening is crucial for identifying individuals at risk of fractures and implementing preventive measure. Dual-energy X-ray absorptiometry (DXA), typically conducted on the lumbar spine and hip bones, is the current gold standard for BMD assessment and serves as the primary tool for osteoporosis diagnosis [1,5,11,12,13]. However, the limited availability of the scanners and their relatively high cost have limited more widespread adoption in screening and post-treatment monitoring [12,13]. Alternatively, calcaneal quantitative ultrasound (QUS) is another common method for assessing BMD [14]. While less expensive and portable compared to DXA scans, QUS generally exhibits lower accuracy [15,16,17,18,19]. A previous study has indicated a sensitivity of 70% and specificity of 73% for QUS in predicting osteoporosis of the lumbar spine when compared with DXA [20]. Another study in the Taiwanese population revealed that the sensitivity and specificity of QUS when compared with DXA are 67.2% and 64.9%, respectively [19]. As a result, a significant portion of the population remains undiagnosed, highlighting the need for improved screening strategies.

The emergence of artificial intelligence (AI) offers promising opportunities to overcome these challenges and improve osteoporosis screening. Several studies have explored the use of AI for opportunistic screening of osteoporosis, which aims at using medical images already acquired for other indications to screen for osteoporosis [21]. This concept enables an increase in screening rates without adding to radiation exposure, costs, or time. Of these studies, research groups have focused on applying deep learning to analyze hand and wrist X-ray images [22], chest X-ray (CXR) images [23,24,25,26,27,28], lumbar X-ray images [29,30], and pelvic X-ray images [29,31,32] for BMD prediction and osteoporosis screening. These studies demonstrate the potential of AI to improve screening efficiency and accuracy. Nevertheless, none of these AI models have been validated in a pivotal clinical study approved for regulatory clearance by national regulatory agencies. In this study, we present the development of VeriOsteo^TM^ OP (Acer Medical Inc., New Taipei City, Taiwan), an AI-assisted screening software (version 1.00.3000) that uses deep learning to analyze the thoracolumbar region (T12–L1) of CXR images for BMD abnormality. We further present the results of the pivotal study that validated the performance of VeriOsteo^TM^ OP, now approved by Taiwan Food and Drug Administration (TFDA) and commercially available in Taiwan as a Class II medical device, by comparing the results with DXA at a medical center and a community clinic.

## 2. Materials and Methods

This study was approved by the Institutional Review Board at the Taichung Veterans General Hospital (TCVGH, https://www.vghtc.gov.tw (accessed on 1 May 2024)), Taichung, Taiwan (IRB Nos: CE21372A for the AI model development, SE23143B for the clinical validation). It was conducted in accordance with the principles of the Declaration of Helsinki and was performed in accordance with current scientific guidelines. The requirement for informed patient consent was waived because the data used were fully de-identified to protect patient confidentiality.

### 2.1. Imaging and Data Collection

In the AI model development stage, the study data for developing the current product model were sourced from the medical database of TCVGH, a medical center in central Taiwan. The study population consisted of 5122 cases with paired CXR images and lumbar spine DXA reports from 1 January 2016 to 31 December 2020 and were aged 20–98 years on the chest index examination date. The data were de-identified and de-linked to ensure anonymity and privacy. Each CXR image was captured under standard clinical conditions following the protocol for posteroanterior (PA) view CXR imaging with a resolution of 1024 × 1024 pixels or higher. Additionally, each image underwent quality assurance by a board-certified orthopedic specialist with over twenty years of experience, ensuring image quality, confirming the presence of complete imaging of the target areas such as the last thoracic vertebra and the first lumbar vertebra, and ensuring no hardware retention or severe deformity in that area. Included participants had DXA bone density reports for lumbar vertebrae taken within 6 months before or after the CXR imaging. The lumbar spine DXA report should include BMD measurement value and T-scores for individual L1, L2, L3, L4 vertebrae. Additionally, each T-score difference between adjacent vertebrae should be ≤1. If participants have multiple CXR images and DXA reports, we select data pairs with the closest temporal difference in their acquisition times. The 5122 cases were randomly allocated into the training, validation, and test sets using simple random sampling, where each case had an equal probability of selection, and sampling was performed without replacement. The training set comprises 4188 pairs of data, the validation set consists of 400 pairs, and the test set comprises 534 pairs of data.

In the clinical validation stage, we collected 440 CXR and DXA paired data from both the TCVGH and Joy Clinic, including 304 pared data from TCVGH and 136 paired data from Joy Clinic.

### 2.2. BMD Measurement

Both in TCVGH and Joy Clinic, the lumbar spine DXA scans were conducted using the GE Lunar iDXA system revision 9 (Madison, WI, USA). The scans were analyzed according to guidelines provided by the Taiwan Radiological Society, which were adapted from the International Society for Clinical Densitometry (ISCD). Due to the absence of an international standard reference for lumbar spine BMD, lumbar T/Z-scores were computed utilizing the manufacturer’s reference values (X-ray Bone Densitometer with enCORE v17 software—User Manual). The T-score compares a patient’s bone density to the average peak bone density of a healthy young female adult (ages 20 to 29), expressed in standard deviations from this average. The Z-score compares a patient’s bone density to the average bone density of a healthy individual of the same age and gender, also expressed in standard deviations from this average. For patients under the age of 50, osteoporosis is determined using the Z-score. For patients aged 50 and older, the T-score is used for this determination. The WHO diagnostic category for osteoporosis is defined as a value for BMD 2.5 standard deviations or more below the young adult mean [12].

### 2.3. Image Acquisition and Pre-Processing

Images were acquired from the Picture Archiving and Communication System (PACS) and anonymized before being used in this study. In TCVGH, the CXR images were generated using radiography systems from Siemens Healthineers AG (Forchheim, Germany), FUJIFILM Corporation (Tokyo, Japan), and Canon Medical Systems Corporation (Tochigi, Japan). In Joy Clinic, the CXR images were produced using a radiography system from Konica Minolta (Tokyo, Japan). All acquired images are stored in Digital Imaging and Communications in Medicine (DICOM) standard version 3.0 format. The acquisition module verifies that the image resolution is higher than 1024 × 1024 pixels to preserve essential bone texture details.

The pre-processing stage is to enhance the usability of the acquired images for further diagnostic assessment and analytical procedures. This study employs the contrast limited adaptive histogram equalization (CLAHE) algorithm, a sophisticated method used widely in medical imaging to improve contrast while retaining essential details within the images.

### 2.4. Image Quality Assessment

This study emphasizes the region of the last vertebra of the thoracic spine and the first vertebra of the lumbar spine (typically T12 and L1). Our automated quality assessment procedure for radiographs is conducted by a dedicated spinal detection module, ensuring the inclusion of these critical regions in the X-ray images.

**Figure 1:**
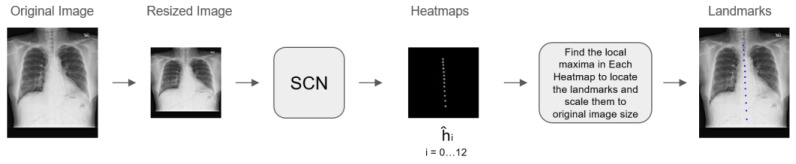
The process from the original image to landmark localization is illustrated. Starting with the original CXR image, the image is resized to fit subsequent processing steps. The resized image is then processed by the Spatial Configuration-Net (SCN) module, which generates heatmaps indicating vertebral locations. Each heatmap corresponds to a specific vertebra, and the local maxima in each heatmap are identified to locate the landmarks. (The network predicts simultaneously 13 heatmaps, i.e., a single heatmap ĥ_i_ for each individual vertebra v_i_. For visualization, the predicted heatmaps are combined into a single image.) These landmarks are then scaled back to the original image size for precise vertebral localization.

**Figure 2:**
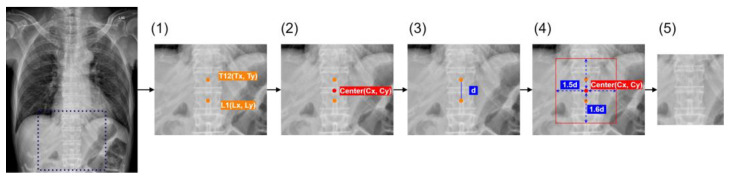
The process of identifying and cropping the T12–L1 region from CXR images. The steps are as follows: (1) Landmark Identification: The T12 and L1 vertebrae are identified on the CXR image; (2) Center Calculation: The center point (Cx, Cy) between T12 and L1 is calculated; (3) Distance Calculation: The distance d between the T12 and L1 points is calculated; (4) ROI Determination: A region of interest (ROI) is defined around the center point. The ROI’s horizontal boundaries are set to 1.5 times d on each side of the center point, and the vertical boundaries are set to 1.6 times d above and below the center point. The defined ROI is cropped from the original CXR image, resulting in a focused image of the T12–L1 region; (5) Cropped Image: The final cropped image containing the T12–L1 region. (The blue box in the leftmost image indicates the range of the area shown in image (1)).

The spinal detection module identifies vertebrae within thoracic cavity X-ray images using the SCN (Spatial Configuration-Net) module, which outputs a heatmap indicating the position of each vertebra (Figure 1). Counting from the top vertebra downwards, the module determines whether the critical vertebrae—12th and 13th vertebra (assumed to be typically T12 and L1 in the content of this report)—are present in the image. Following the localization of these vertebrae, the module segments the image at these specific regions, which are then used for further analysis by a BMD screening AI model (Figure 2).

### 2.5. Algorithm Development

A deep learning algorithm designed to assess the BMD from specified regions of interest (ROIs) was developed. This neural network processes ROI images, which are based on the locations of the last thoracic and first lumbar vertebrae as detailed in Section 2.4 and utilizes fully connected layers with ReLU activation functions to estimate the BMD. During our initial testing phase, we evaluated several backbone networks such as VGG-16, DenseNet-121, ResNet-50, and EfficientNetV2S using data from a pilot study with 2002 training samples. We used the Area Under the Curve (AUC) as the primary metric for model selection. The AUC results were as follows: VGG-16 (0.89), ResNet-50 (0.90), EfficientNetV2S (0.90), and DenseNet-121 (0.92). DenseNet-121 was determined to be the most effective at predicting spinal BMD. Given the robust performance of the model when solely utilizing image-based features, DenseNet-121 was chosen as our primary backbone network for further development of the model. The output of the model consists of the estimated BMD values for vertebrae L1–L4. In the training phase, the ROI is subject to random affine transformations and resized to a resolution of 512 × 512 pixels. The training loss is calculated using the mean square error (MSE) between the BMD values predicted by the model and those measured by DXA scans.

### 2.6. Clinical Validation

In the clinical validation stage, we also validated the model using a clinical dataset collected retrospectively from the databases of TCVGH between 2021 and 2022 and Joy Clinic, a community-based multi-clinic, between 2012 and 2021. We initially screened 1027 cases with lumbar DXA scans, comprising 507 scans from TCVGH and 520 scans from Joy Clinic. These scans were then filtered based on a T-score difference > 1 between L2-L1, L3-L2, and L4-L3. After filtering, 706 DXA scans remained, with 331 from TCVGH and 375 from Joy Clinic. Subsequently, we screened CXR images taken within 6 months of the same case ID (de-identified) for the remaining DXA scans. The CXR images that matched with DXA data within 6 months were further filtered based on image view (PA view) and resolution (1024 × 1024 pixels or higher). The remaining CXR images underwent additional filtering by an experienced orthopedic physician to exclude images without the last thoracic vertebra and the first lumbar vertebra, as well as those with implants in these vertebrae. Following confirmation by the orthopedic physician, 440 eligible CXR images were matched with DXA scans. Among these, 304 DXA data were from TCVGH, and 136 were from Joy Clinic.

### 2.7. Evaluation of BMD Prediction Performance and Statistics

Evaluation of all performance measures was performed on the test dataset in the model development phase and the clinical validation dataset. Suspected abnormal BMD (saBMD) is defined as T-score ≤ −2.5 for cases aged ≥ 50 years old or Z-score ≤ −2.0 for cases aged < 50 years old. On the contrary, non-suspected abnormal BMD (non-saBMD) is defined as T-score > −2.5 for cases aged ≥ 50 years old or Z-score > −2.0 for cases aged < 50 years old. The overall discriminative ability to discern saBMD individuals was evaluated using the methodology of area under receiver operating characteristic curve (AUROC). Other measures were also calculated, including accuracy, sensitivity, specificity, positive predictive value (PPV), and negative predictive value (NPV). The scatter plot visualized the agreement between predicted and measured BMD scores, and Pearson’s correlation coefficient was calculated. For the demographic comparison between the populations from TCVGH and Joy Clinic, means were compared using Student’s *t*-test and categorical variables were compared using a Chi-square test. For the comparison of the clinical performance of VeriOsteo^TM^ OP (Acer Medical Inc., New Taipei City, Taiwan) at TCVGH and Joy Clinic, a two-proportion z-test was used. Two-sided *p*-values are reported throughout the manuscript.

## 3. Results

### 3.1. The Design and Workflow of VeriOsteo^TM^ OP

**Figure 3:**
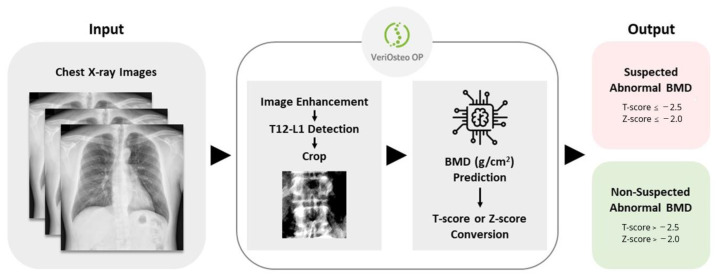
Schematic representation of the workflow of VeriOsteo^TM^ OP.

A schematic representation of the workflow of VeriOsteo^TM^ OP is shown in Figure 3. VeriOsteo^TM^ OP takes CXR images as the input. It then enhances the images (using CLAHE), detects the T12–L1 region of the spine, and crops the T12–L1 region for analysis. The T12–L1 region is resized to 512 × 512 pixels before being input into the AI model for analysis. By analyzing the T12–L1 region of the spine, the AI model predicts the BMD and converts the BMD to a T-score (for cases aged ≥ 50 years old) or Z-score (for cases aged < 50 years old). It then categorizes the results into saBMD or non-saBMD as the output.

### 3.2. Characteristics of the Training/Pre-Clinical Dataset

The characteristics of the training/pre-clinical dataset are listed in Table 1. Of the 4,188 cases (3263 women [78%], mean age, 64.2 [SD, 13.1] years) for training, 3,731 (89%) were 50 years and over and 457 (11%) were under 50 years old. The mean BMD was 1.01 (SD, 0.20) and the mean T-score and Z-score were −1.44 (SD, 1.69) and 0.62 (SD, 1.59), respectively. In terms of BMD categories, 1129 (27%) were saBMD (positive) and 3059 (73%) were non-saBMD (negative). Of the 400 cases (326 women [82%], mean age, 62.1 [SD, 13.8] years) for validation, 334 (84%) were 50 years and over and 66 (17%) were under 50 years old. The mean BMD was 0.99 (SD, 0.18) and the mean T-score and Z-score were −1.57 (SD, 1.53) and 0.34 (SD, 1.42), respectively. In terms of BMD categories, 108 (27%) were saBMD (positive) and 292 (73%) were non-saBMD (negative). Of the 534 cases (437 women [82%], mean age, 60.7 [SD, 13.1] years) for pre-clinical test, 435 (81%) were 50 years and over and 99 (19%) were under 50 years old. The mean BMD was 0.99 (SD, 0.18) and the mean T-score and Z-score were −1.61 (SD, 1.53) and 0.26 (SD, 1.41), respectively. In terms of BMD categories, 152 (28%) were saBMD (positive) and 382 (72%) were non-saBMD (negative).

### 3.3. Pre-Clinical Performance of VeriOsteo^TM^ OP

**Figure 4:**
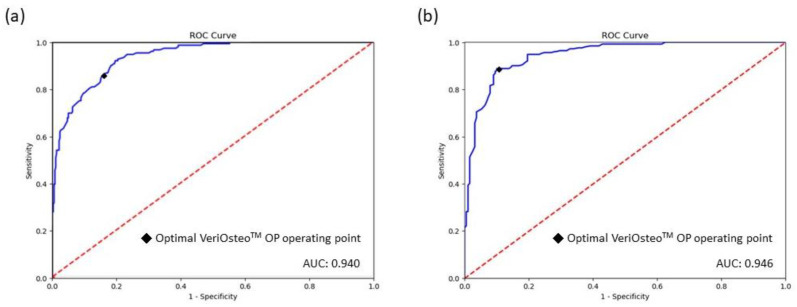
Receiver operating characteristic curves and the areas under the curve of the VeriOsteo^TM^ OP model for screening of saBMD: (**a**) in the pre-clinical test set; (**b**) in the clinical validation set.

**Figure 5:**
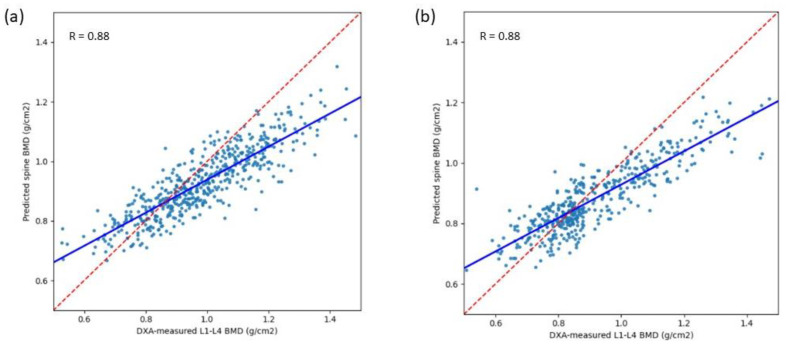
Scatter plot and the Pearson’s correlation coefficient for predicted/measured BMD for (**a**) pre-clinical test; (**b**) clinical validation. (Red dashed line: Identity line, Blue line: Least squares regression line).

The pre-clinical performance of VeriOsteo^TM^ OP is summarized in Table 2. For detecting saBMD, the algorithm of VeriOsteo^TM^ OP achieved an AUC of 0.940 (95% CI: 0.923–0.957) (Figure 4a). At the optimal operating point, the algorithm had a sensitivity of 86.2% (95% CI: 79.7–91.2%) and a specificity of 83.8% (95% CI: 79.7–87.3%). The overall accuracy was 84.5% (95% CI: 81.1–87.4%). The positive and negative predictive values (PPV and NPV) were 67.9% (95% CI: 62.5–72.8%) and 93.8% (95% CI: 91.1–95.8%), respectively. The model exhibited good linear correlation of predicted BMD with regard to measured BMD as depicted in the scatter plot in Figure 5a. The Pearson’s correlation coefficient between DXA-measured and model-predicted BMD was 0.88.

### 3.4. Characteristics of the Clinical Validation Dataset

The characteristics of the clinical validation dataset are listed in Table 1. Of the 440 cases enrolled, 304 were from TCVGH and 136 were from Joy Clinic. Of the 304 cases (250 women [82%], mean age, 63.5 [SD, 12.5] years) from TCVGH, 270 (89%) were 50 years and over and 34 (11%) were under 50 years old. The mean BMD was 0.90 (SD, 0.18) and the mean T-score and Z-score were −2.34 (SD, 1.49) and −0.30 (SD, 1.29), respectively. In terms of BMD categories, 184 (61%) were saBMD (positive) and 120 (39%) were non-saBMD (negative). In terms of image quality, four (1%) images from this group were not evaluable. Of the 136 cases (101 women [74%], mean age, 60.1 [SD, 11.9] years) from Joy Clinic, 106 (78%) were 50 years and over and 30 (22%) were under 50 years old. The mean BMD was 0.95 (SD, 0.18) and the mean T-score and Z-score were −1.89 (SD, 1.48) and −0.06 (SD, 1.13), respectively. In terms of BMD categories, 69 (51%) were saBMD (positive) and 67 (49%) were non-saBMD (negative). No image from this group was not evaluable. Overall, of the total of 440 cases enrolled (351 women [80%], mean age, 62.5 [SD, 12.4] years), 376 (85%) were 50 years and over and 64 (15%) were under 50 years old. The mean BMD was 0.92 (SD, 0.18) and the mean T-score and Z-score were −2.20 (SD, 1.50) and −0.23 (SD, 1.25), respectively. In terms of BMD categories, 253 (58%) were saBMD (positive) and 187 (43%) were non-saBMD (negative). When comparing the demographics between TCVGH and Joy Clinic, the mean age, percentage of cases 50 years and over, the mean BMD, and the mean T-score were significantly different (*p*-value ≤ 0.05). But the percentage of female cases (*p*-value = 0.0544) and the mean Z-score (*p*-value = 0.0619) were not significantly different between the two subgroups.

### 3.5. Clinical Validation of the Performance of VeriOsteo^TM^ OP

The clinical validation performance of VeriOsteo^TM^ OP is summarized in Table 2. When combining the performance results from the TCVGH and the Joy Clinic groups for detecting saBMD, the algorithm of VeriOsteo^TM^ OP achieved an AUC of 0.946 (95% CI: 0.925–0.967) (Figure 4b). At the optimal operating point, the algorithm had a sensitivity of 88.7% (95% CI: 84.1–92.4%) and a specificity of 89.4% (95% CI: 84.1–93.4%). The overall accuracy was 89.0% (95% CI: 85.7–91.8%). The positive and negative predictive values (PPV and NPV) were 91.7% (95% CI: 87.4–94.8%) and 85.7% (95% CI: 80.0–90.3%), respectively. The model remained robust with good predictive performance of predicted BMD with regard to measured BMD as depicted in the scatter plot in Figure 5b. The Pearson’s correlation coefficient between DXA-measured and model-predicted BMD was 0.88. When comparing the performance between the two subgroups (TCVGH vs. Joy Clinic), the AUC, accuracy, sensitivity, specificity, PPV, and Pearson’s correlation coefficient were not significantly different (*p*-value > 0.05). Only NPV (*p*-value = 0.252) was significantly different between TCVGH and Joy Clinic.

## 4. Discussion

Osteoporosis is a silent disease characterized by low bone mineral density (BMD) that especially affects the elderly [2,5]. Due to the aging population, global osteoporosis prevalence is on the rise. According to data from the National Health Insurance system in Taiwan, the prevalence of osteoporosis among individuals aged 50 and over has increased significantly from 17.4% in 2001 to 25.0% in 2011 [33]. Since osteoporosis is difficult to reverse, it is imperative to detect the disease early so that preventive measures can be taken to ameliorate the deterioration. However, the screening rate is in general low even in developed countries [34]. This is mostly due to the disease’s lack of symptoms, lack of awareness, and accessibility issues such as limited access to DXA scans. DXA scans can be limited in availability, expensive, time-consuming, and involve radiation exposure [35,36]. As a result, expansion to population-based screening would be difficult to realize.

With the recent advancements in deep machine learning, many researchers have proposed the idea of opportunistic screening using already acquired images as a potential solution to expand screening to a wider population. Study on vertebral body fractures analysis on computed tomography (CT) utilizing AI models has yielded reliable results [37]. Typically, CT images are taken when the patient is undergoing treatment. There is potential to advance the timing of osteoporosis screening. Several groups have developed AI-based algorithms that analyze X-ray images of hand and wrist [22], lumbar spine [29], and pelvis [29,31,32] to predict BMD. However, X-ray images from these regions are generally not widely available. Therefore, when these solutions are implemented into the clinical setting, patients may need to take a new X-ray image for the AI model to analyze, which defeats the purpose of “opportunistic” screening. Here, we strategically chose CXR images as the model input because this image modality widely used for various medical purposes and easily accessible from routine health checks, especially in Asia. We further focus on the T12–L1 as the region of interest as these are the vertebrae where compression fracture is likely to occur. The VeriOsteo^TM^ OP model was trained with the lumbar spine L1–L4 DXA results as the gold standard reference, which is a clinically relevant measurement for the diagnosis of osteoporosis. In comparison, VeriOsteo^TM^ OP correlated well with the gold standard DXA-measured BMD in both pre-clinical and clinical validation sets with good performance to screen for saBMD (AUC = 0.940 and 0.946, respectively). Furthermore, in our clinical validation, when compared with similar AI models that analyze CXRs to predict lumbar BMD for the screening of osteoporosis [23,24,26,27,28], VeriOsteo^TM^ OP exhibited the highest correlation (R = 0.88) and its performance was either comparable or superior in terms of AUC (0.946) and overall accuracy (89.0%). Since VeriOsteo^TM^ OP is intended to be used for preliminary screening purposes, we selected the optimal operating point so that the model achieved a well-balanced level of sensitivity (88.7%), specificity (89.4%), PPV (91.7%), and NPV (85.7%). From the public health point of view, with a relatively high prevalence of osteoporosis among the elderly, the high PPV is of particular importance to minimize an excessive number of false positives.

In the clinical validation, we selected two validation sites of different scale. TCVGH is a large-scale comprehensive medical center in the central regions of Taiwan while Joy Clinic is a smaller-scale community clinic focusing on chronic diseases. In our subgroup analysis of the patients enrolled from these two sites, we found that the demographics of the two populations from these subgroups were mostly significantly different. Nevertheless, when comparing the performance of VeriOsteo^TM^ OP for detecting saBMD validated using datasets from these two sites, we found that the AUC, accuracy, sensitivity, specificity, PPV, and Pearson’s correlation coefficient were not significantly different. These results demonstrate that the performance of VeriOsteo^TM^ OP is robust when the tool is applied to different clinical institutions. The robustness of the performance of such AI-based screening tools is critical to their subsequent wider adoption towards the concept of population-based screening.

Despite the careful design of the development and clinical validation of VeriOsteo^TM^ OP, this study had several limitations. First, our training/pre-clinical dataset was solely from TCVGH, which is a medical center that tends to have a more complex population with a large proportion of patients with severe diseases. But when VeriOsteo^TM^ OP was validated using data from Joy Clinic, an external site with presumably patients with fewer complications, the performance was comparable to that validated using data from TCVGH. However, as mentioned earlier, the radiography systems used for CXR acquisition are from Siemens Healthineers AG, FUJIFILM Corporation, and Canon Medical Systems, which are different from the Konica Minolta system used in Joy Clinic. In the future, the model is expected to be further optimized and become even more robust when more data from a healthier population are added to fine-tune the model. Second, the sample sizes of both the training/pre-clinical and clinical validation datasets are relatively small. As with many other medical imaging-based projects, data availability is always a concern. The emergence of foundation models offers a significant opportunity in addressing the problem using minimal amounts of labeled data [38] during training. Applying such technology to our future generations of models has the potential to make the models robust across different real-world healthcare settings with diverse populations. Validations using real-world data are also planned as VeriOsteo^TM^ OP is being deployed into real clinical settings. Third, in all the datasets used in this study, female patients and patients 50 years and over comprise the majority (over 70%) of the populations. As osteoporosis is associated with gender and age, such a composition reflects the prevalence trend in the real world. However, since only 64 patients under 50 years old were enrolled in the clinical validation with limited positive (saBMD) cases, the final VeriOsteo^TM^ OP model approved by TFDA was limited to subjects 50 years old or over. Once more data of patients under 50 years old are available, the model can further be fine-tuned to be applied to this younger population. Fourth, the data collected and tested in this study are based on the population in Taiwan, which is known for the lack of ethnical diversity. The performance of the AI model across other ethnicities is yet to be determined. Finally, the ground truth of both the model training phase and the clinical validation study was based on BMD measurements from a single brand and model of DXA scanner. The effect of inter-model variation on predicted BMD and T-score needs to be considered in future validation studies.

This study presented a robust opportunistic screening tool, VeriOsteo^TM^ OP, that utilizes AI to analyze CXR images for saBMD. It is the first AI-based software (version 1.00.3000) as a medical device (SaMD) of its kind to have been approved by a national regulatory agency. The implementation of such an AI-based tool has the potential to improve the efficiency and accessibility of osteoporosis screening, ultimately contributing to earlier diagnosis, better patient outcomes, and even lower incidence of osteoporosis-related fractures. Some of the advantages of AI-based screening include lower cost, wider accessibility, and integration into existing healthcare workflows. The deployment of VeriOsteo^TM^ OP into real clinical settings is expected to revolutionize the current practice of osteoporosis screening by offering preliminary screening to a broader population.

## Figures and Tables

**Table 1:** Image characteristics of the training and clinical validation datasets.

	Training/Pre-Clinical Dataset			Clinical Validation Dataset			
	Training	Validation	Test	TCVGH	Joy Clinic	Overall	*p*-Value *
Number, n	4188	400	534	304	136	440	–
Demographics							
Female, n (%)	3263 (78)	326 (82)	437 (82)	250 (82)	101 (74)	351 (80)	0.0544
Male, n (%)	925 (22)	74 (18)	97 (18)	54 (18)	35 (26)	89 (20)	
Age (years), mean ± SD	64.2 ± 13.1	62.1 ± 13.8	60.7 ± 13.1	63.5 ± 12.5	60.1 ± 11.9	62.5 ± 12.4	0.0077
Age ≥ 50 years old, n (%)	3731 (89)	334 (84)	435 (81)	270 (89)	106 (78)	376 (85)	0.0028
Age < 50 years old, n (%)	457 (11)	66 (17)	99 (19)	34 (11)	30 (22)	64 (15)	
Bone Mass Density							
BMD (g/cm^2^), mean ± SD	1.01 ± 0.20	0.99 ± 0.18	0.99 ± 0.18	0.90 ± 0.18	0.95 ± 0.18	0.92 ± 0.18	0.0074
T-score, mean ± SD	−1.44 ± 1.69	−1.57 ± 1.53	−1.61± 1.53	−2.34 ± 1.49	−1.89 ± 1.48	−2.20 ± 1.50	0.0035
Z-score, mean ± SD	0.62 ± 1.59	0.34 ± 1.42	0.26 ± 1.41	−0.30 ± 1.29	−0.06 ± 1.13	−0.23 ± 1.25	0.0619
saBMD ^1^, n (%)	1129 (27)	108 (27)	152 (28)	184 (61)	69 (51)	253 (58)	–
Non-saBMD ^2^, n (%)	3059 (73)	292 (73)	382 (72)	120 (39)	67 (49)	187 (43)	–
Collected images, n (%)							
Evaluable ^3^	4188 (100)	400 (100)	534 (100)	300 (99)	136 (100)	436 (99)	–
Not evaluable ^4^	–	–	–	4 (1)	0 (0)	4 (1)	–

* *p*-value between the Taichung Veterans General Hospital (TCVGH) group and the Joy Clinic group in the clinical validation dataset. ^1^ saBMD: suspected abnormal bone mineral density. ^2^ Non-saBMD: non-suspected abnormal bone mineral density. ^3^ VeriOsteo^TM^ OP included two models. One is the spinal detection module. The other is the BMD screening AI model. This represents the quantity of evaluable CXR by the spinal detection module. ^4^ The number of CXRs excluded by the spinal detection module.

**Table 2:** Pre-clinical and clinical performance of screening for saBMD by VeriOsteo^TM^ OP.

	Pre-Clinical Test	Clinical Validation			
	Testing	TCVGH	Joy Clinic	Overall	*p*-Value *
AUC (95% CI)	0.940 (0.923–0.957)	0.948 (0.924–0.972)	0.938 (0.895–0.980)	0.946 (0.925–0.967)	0.6901
Accuracy (%) (95% CI)	84.5 (81.1–87.4)	88.3 (84.2–91.7)	90.4 (84.2–94.8)	89.0 (85.7–91.8)	0.5148
Sensitivity (%) (95% CI)	86.2 (79.7–91.2)	86.7 (80.9–91.3)	94.0 (85.4–98.4)	88.7 (84.1–92.4)	0.1072
Specificity (%) (95% CI)	83.8 (79.7–87.3)	90.8 (84.1–95.3)	87.0 (76.7–93.9)	89.4 (84.1–93.4)	0.4154
PPV (%) (95% CI)	67.9 (62.5–72.8)	93.5 (88.6–96.7)	87.5 (77.6–94.1)	91.7 (87.4–94.8)	0.1263
NPV (%) (95% CI)	93.8 (91.1–95.8)	81.8 (74.2–88.0)	93.8 (84.8–98.3)	85.7 (80.0–90.3)	0.0252
Pearson’s correlation coefficient (95% CI)	0.88 (0.86–0.90)	0.88 (0.85–0.90)	0.88 (0.84–0.91)	0.88 (0.86–0.90)	0.8298

* *p*-value between the Taichung Veterans General Hospital (TCVGH) group and the Joy Clinic group in the clinical validation dataset.

## Data Availability

The data presented in this study are available upon considerable request to the corresponding author (Kun-Hui Chen).

## References

[1] Kanis J.A. (2002). Diagnosis of Osteoporosis and Assessment of Fracture Risk. Lancet.

[2] Sözen T., Özışık L., Başaran N.Ç. (2017). An Overview and Management of Osteoporosis. Eur. J. Rheumatol..

[3] (1993). Consensus Development Conference: Diagnosis, Prophylaxis, and Treatment of Osteoporosis. Am. J. Med..

[4] Kanis J.A. (1994). Assessment of Fracture Risk and Its Application to Screening for Postmenopausal Osteoporosis: Synopsis of a WHO Report. WHO Study Group. Osteoporos. Int..

[5] Johnston C.B., Dagar M. (2020). Osteoporosis in Older Adults. Med. Clin. N. Am..

[6] LeBoff M.S., Greenspan S.L., Insogna K.L., Lewiecki E.M., Saag K.G., Singer A.J., Siris E.S. (2022). The Clinician’s Guide to Prevention and Treatment of Osteoporosis. Osteoporos. Int..

[7] Siris E.S., Chen Y.-T., Abbott T.A., Barrett-Connor E., Miller P.D., Wehren L.E., Berger M.L. (2004). Bone Mineral Density Thresholds for Pharmacological Intervention to Prevent Fractures. Arch. Intern. Med..

[8] Li G., Thabane L., Papaioannou A., Ioannidis G., Levine M.A.H., Adachi J.D. (2017). An Overview of Osteoporosis and Frailty in the Elderly. BMC Musculoskelet. Disord..

[9] Melton L.J., Chrischilles E.A., Cooper C., Lane A.W., Riggs B.L. (1992). Perspective. How Many Women Have Osteoporosis?. J. Bone Miner. Res..

[10] Health Promotion Administration, Ministry of Health and Welfare, National Health Research Institutes, The Taiwanese Osteoporosis Association Osteoporosis Clinical Treatment. https://www.hpa.gov.tw/Pages/List.aspx?nodeid=1151.

[11] Nayak S., Edwards D.L., Saleh A.A., Greenspan S.L. (2015). Systematic Review and Meta-Analysis of the Performance of Clinical Risk Assessment Instruments for Screening for Osteoporosis or Low Bone Density. Osteoporos. Int..

[12] Dimai H.P. (2017). Use of Dual-Energy X-ray Absorptiometry (DXA) for Diagnosis and Fracture Risk Assessment; WHO-Criteria, T- and Z-Score, and Reference Databases. Bone.

[13] De Oliveira M.A., Moraes R., Castanha E.B., Prevedello A.S., Vieira Filho J., Bussolaro F.A., García Cava D. (2022). Osteoporosis Screening: Applied Methods and Technological Trends. Med. Eng. Phys..

[14] Hans D., Baim S. (2017). Quantitative Ultrasound (QUS) in the Management of Osteoporosis and Assessment of Fracture Risk. J. Clin. Densitom..

[15] Njeh C.F., Hans D., Li J., Fan B., Fuerst T., He Y.Q., Tsuda-Futami E., Lu Y., Wu C.Y., Genant H.K. (2000). Comparison of Six Calcaneal Quantitative Ultrasound Devices: Precision and Hip Fracture Discrimination. Osteoporos. Int..

[16] Ng D.C., Sundram F.X. (1998). Bone Mineral Density–Correlation between Quantitative Ultrasound Characteristics and Dual Energy X-ray Absorptiometry. Ann. Acad. Med. Singap..

[17] Villa P., Lassandro A.P., Moruzzi M.C., Amar I.D., Vacca L., Di Nardo F., De Waure C., Pontecorvi A., Scambia G. (2016). A Non-Invasive Prevention Program Model for the Assessment of Osteoporosis in the Early Postmenopausal Period: A Pilot Study on FRAX^®^ and QUS Tools Advantages. J. Endocrinol. Investig..

[18] Fenton J.J., Deyo R.A. (2003). Patient Self-Referral for Radiologic Screening Tests: Clinical and Ethical Concerns. J. Am. Board. Fam. Pract..

[19] Yen C.-C., Lin W.-C., Wang T.-H., Chen G.-F., Chou D.-Y., Lin D.-M., Lin S.-Y., Chan M.-H., Wu J.-M., Tseng C.-D. (2021). Pre-Screening for Osteoporosis with Calcaneus Quantitative Ultrasound and Dual-Energy X-ray Absorptiometry Bone Density. Sci. Rep..

[20] Dubois E.F., van den Bergh J.P., Smals A.G., van de Meerendonk C.W., Zwinderman A.H., Schweitzer D.H. (2001). Comparison of Quantitative Ultrasound Parameters with Dual Energy X-ray Absorptiometry in Pre- and Postmenopausal Women. Neth. J. Med..

[21] Yen T.-Y., Ho C.-S., Chen Y.-P., Pei Y.-C. (2024). Diagnostic Accuracy of Deep Learning for the Prediction of Osteoporosis Using Plain X-rays: A Systematic Review and Meta-Analysis. Diagnostics.

[22] Areeckal A.S., Jayasheelan N., Kamath J., Zawadynski S., Kocher M., David S.S. (2018). Early Diagnosis of Osteoporosis Using Radiogrammetry and Texture Analysis from Hand and Wrist Radiographs in Indian Population. Osteoporos. Int..

[23] Wang F., Zheng K., Wang Y., Zhou X., Lu L., Xiao J., Wu M., Kuo C.-F., Miao S., Rekik I., Adeli E., Park S.H., Schnabel J. (2021). Opportunistic Screening of Osteoporosis Using Plain Film Chest X-ray.

[24] Jang M., Kim M., Bae S.J., Lee S.H., Koh J.-M., Kim N. (2022). Opportunistic Osteoporosis Screening Using Chest Radiographs with Deep Learning: Development and External Validation with a Cohort Dataset. J. Bone Miner. Res..

[25] Asamoto T., Takegami Y., Sato Y., Takahara S., Yamamoto N., Inagaki N., Maki S., Saito M., Imagama S. (2024). External Validation of a Deep Learning Model for Predicting Bone Mineral Density on Chest Radiographs. Arch. Osteoporos..

[26] Sato Y., Yamamoto N., Inagaki N., Iesaki Y., Asamoto T., Suzuki T., Takahara S. (2022). Deep Learning for Bone Mineral Density and T-Score Prediction from Chest X-rays: A Multicenter Study. Biomedicines.

[27] Tsai D.-J., Lin C., Lin C.-S., Lee C.-C., Wang C.-H., Fang W.-H. (2024). Artificial Intelligence-Enabled Chest X-ray Classifies Osteoporosis and Identifies Mortality Risk. J. Med. Syst..

[28] Wang F., Zheng K., Lu L., Xiao J., Wu M., Kuo C.-F., Miao S. (2023). Lumbar Bone Mineral Density Estimation from Chest X-ray Images: Anatomy-Aware Attentive Multi-ROI Modeling. IEEE Trans. Med. Imaging.

[29] Hsieh C.-I., Zheng K., Lin C., Mei L., Lu L., Li W., Chen F.-P., Wang Y., Zhou X., Wang F. (2021). Automated Bone Mineral Density Prediction and Fracture Risk Assessment Using Plain Radiographs via Deep Learning. Nat. Commun..

[30] Zhang B., Yu K., Ning Z., Wang K., Dong Y., Liu X., Liu S., Wang J., Zhu C., Yu Q. (2020). Deep Learning of Lumbar Spine X-ray for Osteopenia and Osteoporosis Screening: A Multicenter Retrospective Cohort Study. Bone.

[31] Ho C.-S., Chen Y.-P., Fan T.-Y., Kuo C.-F., Yen T.-Y., Liu Y.-C., Pei Y.-C. (2021). Application of Deep Learning Neural Network in Predicting Bone Mineral Density from Plain X-ray Radiography. Arch. Osteoporos..

[32] Kim S., Kim B.R., Chae H.-D., Lee J., Ye S.-J., Kim D.H., Hong S.H., Choi J.-Y., Yoo H.J. (2022). Deep Radiomics-Based Approach to the Diagnosis of Osteoporosis Using Hip Radiographs. Radiol. Artif. Intell..

[33] Tai T.-W., Huang C.-F., Huang H.-K., Yang R.-S., Chen J.-F., Cheng T.-T., Chen F.-P., Chen C.-H., Chang Y.-F., Hung W.-C. (2023). Clinical Practice Guidelines for the Prevention and Treatment of Osteoporosis in Taiwan: 2022 Update. J. Formos. Med. Assoc..

[34] Choksi P., Gay B.L., Haymart M.R., Papaleontiou M. (2023). Physician-Reported Barriers to Osteoporosis Screening: A Nationwide Survey. Endocr. Pract..

[35] Pisani P., Renna M.D., Conversano F., Casciaro E., Muratore M., Quarta E., Paola M.D., Casciaro S. (2013). Screening and Early Diagnosis of Osteoporosis through X-ray and Ultrasound Based Techniques. World J. Radiol..

[36] Marín F., López-Bastida J., Díez-Pérez A., Sacristán J.A., ECOSAP DXA Substudy Group Investigators (2004). Bone Mineral Density Referral for Dual-Energy X-ray Absorptiometry Using Quantitative Ultrasound as a Prescreening Tool in Postmenopausal Women from the General Population: A Cost-Effectiveness Analysis. Calcif. Tissue Int..

[37] Constanze P., Eren Y., Carsten M., Hyungseok J., Olav J., Cristian L., Christian B., Claus C.G., Sam S. (2024). AI-based automated detection and stability analysis of traumatic vertebral body fractures on computed tomography. Eur. J. Radiol..

[38] Azizi S., Culp L., Freyberg J., Mustafa B., Baur S., Kornblith S., Chen T., Tomasev N., Mitrović J., Strachan P. (2023). Robust and Data-Efficient Generalization of Self-Supervised Machine Learning for Diagnostic Imaging. Nat. Biomed. Eng..

